# Cognitive decline among older adults: A hidden preexisting condition and its role in ‘brain‐at‐risk’ surgical patients

**DOI:** 10.1002/brb3.2095

**Published:** 2021-03-04

**Authors:** Connor T. A. Brenna, Beverley A. Orser, Sinziana Avramescu, Andrew Fleet, Lilia Kaustov, Stephen Choi

**Affiliations:** ^1^ Department of Medicine University of Toronto Toronto ON Canada; ^2^ Department of Anesthesia Sunnybrook Health Sciences Centre Toronto ON Canada; ^3^ Department of Physiology University of Toronto Toronto ON Canada; ^4^ Department of Anesthesiology and Pain Medicine University of Toronto Toronto ON Canada; ^5^ Department of Anesthesia Humber River Hospital Toronto ON Canada

**Keywords:** anesthesia, cognitive assessment screening instrument, mild neurocognitive disorder, neurocognitive tests, postoperative cognitive disorder

## Abstract

Preexisting cognitive impairment is an important, but underrecognized, predictor of postoperative neurocognitive dysfunction, a common and important sequela of surgery. We have applied computerized neuropsychological testing as an efficient and reliable means of detecting preexisting cognitive impairment in two studies of cardiac and noncardiac surgical populations and propose that this tool has great potential in routine clinical diagnosis.

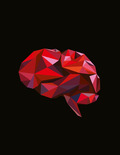

## 1 PERIOPERATIVE NEUROCOGNITIVE DISORDERS

Perioperative neurocognitive disorders (PNDs) comprise a family of cognitive dysfunctions related to anesthesia and surgery. Among these are several common surgical sequelae: postoperative delirium (PD), delayed neurocognitive recovery (dNCR), and postoperative neurocognitive disorder (P‐NCD) (Evered et al., 2018). P‐NCD manifests as a persistent decrease in cognitive ability relative to preoperative baseline levels (in contrast to PD and dNCR which, by definition, resolve within 7 and 30 days, respectively). The diagnosis of PND has three core elements: a subjective cognitive complaint from a patient, caregiver, or clinician; objective impairment of performance on a cognitive test, defined by standard deviations below norms or controls (mild and major impairment are delineated as a decrease of one to two standard deviations versus two or more standard deviations, respectively); and a declining in function, particularly with respect to instrumental activities of daily living (Evered et al., 2018).

While P‐NCD can affect any patient after surgery, its incidence and consequences are highest among older adults with rates estimated to be between 10% and 30% at 3 months after surgery, depending on surgical procedure (Knipp et al., 2017; Moller et al., 1998; Monk et al., 2008). P‐NCD significantly impacts both the quality of life and the ability of patients to carry out routine activities of daily living (Ahlgren et al., 2003; Evered et al., 2016; Price et al., 2008; Steinmetz et al., 2009). P‐NCD is also associated with the development of dementia (Lingehall et al., 2017; Lundström et al., 2003; Olofsson et al., 2018; Wang et al., 2020) as well as an increase in the utilization of social assistance, early departure from the labor force, and an overall increase in postoperative mortality (Ahlgren et al., 2003; Evered et al., 2016; Monk et al., 2008; Price et al., 2008; Steinmetz et al., 2009). It therefore represents a considerable human and economic burden.

P‐NCD and dNCR are complex, multifactorial conditions with several identified risk factors including preexisting cognitive impairment (pre‐CI), advanced age, postoperative infection, higher surgical complexity, lower levels of patient education, central nervous system pathology, and chronic inflammatory disease (Daiello et al., 2019; Deiner et al., 2017; Evered & Silbert, 2018; Kadoi et al., 2011). Among these, pre‐CI, which is characterized by a preoperative decrease in cognition relative to healthy, age‐matched controls, is of particular interest because it is highly prevalent and may represent an actionable target for focused early or perioperative intervention.

The vast majority of pre‐CI is undiagnosed, in part because cognitive status is rarely, if ever, assessed preoperatively. Thus, there is an urgent need for validated and efficient preoperative cognitive screening procedures. Indeed, one study of older adults undergoing vascular surgery reported that the incidence of pre‐CI incidence was 68%, and the condition was previously unrecognized in 88.3% of those patients who met the diagnostic criteria (Partridge et al., 2014). Another study of 215 older adults who were undergoing major elective surgery, similarly reported that 121 patients had performance criteria of pre‐CI, yet only two patients had any prior documented diagnosis of cognitive impairment (Smith & Yeow, 2016). The identification of pre‐CI is therefore underrecognized and should be a critical priority in older adult surgical populations, as well as for patients managed beyond the perioperative setting under the purview of geriatricians, internists, and primary care providers.

## 2 IDENTIFYING PREEXISTING COGNITIVE IMPAIRMENT

Baseline cognitive screening to detect pre‐CI prior to surgery has the formidable potential to identify surgical patients who are predisposed to P‐NCD. These patients could benefit considerably from tailored preventative measures. Presently, we are employing a computerized neuropsychological testing tool, the Cogstate Brief Battery (CBB) (Maruff et al., 2013; Mielke et al., 2015), to identify pre‐CI and P‐NCD among surgical patients in a clinical research environment. This approach affords us the unique opportunity to directly assess the predictive utility of CBB to detect pre‐CI and to measure persistent, progressive cognitive decline.

The advantages of computerized neurocognitive testing are considerable and include the low time requirements of clinical staff, standardization, automated data handling and score calculation, and easy accessibility. The tool also transcends the limitations of both language and location. The CBB examines four cognitive domains, comparing them to a large international database (rather than relying on changes in performance between two time points): attention and vigilance, processing speed, working memory, and visual learning (Maruff et al., 2009). The CBB has been validated as a neuropsychological screening tool, in a wide variety of clinical contexts when compared to conventional test batteries (Maruff et al., 2009). These settings include the perioperative environment (Ichimura et al., 2010; Silbert et al. 2004), where CBB was shown to have a comparable sensitivity (80%) and specificity (85%) to the Montreal Cognitive Assessment (MoCA) for the detection of mild NCD (De Roeck et al., 2019; Mielke et al., 2015). The CBB test uses digital representations of regular playing cards, which are drawn at random. The factorial combinations produced from 52 cards create practically infinite alternate forms of the test, minimizing learning and practice effects, which refer to the improved test scores observed with repeated exposures that many neurocognitive tests are vulnerable to (Falleti et al., 2006; Maruff et al., 2013). Additionally, the CBB is reliable in tests of adults across a wide range of ages and computer experience (Mielke et al. 2015). Our primary focus in applying the CBB was on older patients undergoing surgery, especially those at greatest risk of P‐NCD (e.g., those undergoing complex surgery under general anesthetic). To further characterize the relationship between pre‐CI and P‐NCD, we have initiated two major studies: *COGNIGRAM* (observational study, sample size 600, NCT03147937 (Choi et al., 2019)), which includes patients undergoing total hip or knee arthroplasty; and *CODEX* (multisite, randomized control trial, sample size 2,400, NCT04289142) patients undergoing cardiac surgery (a population with the highest risk of developing postsurgical cognitive complications). The ongoing studies will delineate the impact of pre‐CI on perioperative cognitive trajectories in patients undergoing cardiac surgery or patients undergoing joint arthroplasty surgery (a procedure that involves less physiologic stress, but are among the most common major surgical procedures in older patients).

## 3 PRELIMINARY OBSERVATIONS

The primary outcome of the studies is the incidence of major P‐NCD (defined as a decline in cognitive function with a z‐score > 1.96 below age‐matched, nonoperative controls) at 3 and 4.5 months after surgery for *CODEX* and *COGNIGRAM*, respectively. Herein, we report on early preoperative baseline cognition status of patients evaluated for participation. Among the exclusion criteria for each study is the level of baseline major neurocognitive dysfunction. Patient with particularly poor baseline function is ineligible for study participation. Specifically, the low score for exclusion from the study was defined as a Cogstate Brief Battery Score of <80 in one or more of four cognitive domains. This score corresponds to a z‐score of 1.96 using the Reliable Change Index.

The *CODEX* began as a pilot study of patients undergoing cardiac surgery (NCT03480061) and was subsequently expanded to a multicenter trial (NCT04289142). Approximately 31% of cardiac surgery patients who completed baseline CBB testing had major pre‐CI (20 of 64 patients have already been tested, from a projected sample size of 2,400). We observed that the patients were far more likely to exhibit deficits in psychomotor function and attention than in the cognitive domains of learning or working memory. These findings align closely with those of other studies of pre‐CI assessed in older adults who were undergoing cardiac surgery, where the incidence of pre‐CI ranged from 25% to 45% (Hogue et al., 2006; Hudetz et al., 2012; Silbert et al., 2007). Notably, pre‐CI was detected much more frequently in patients who completed baseline CBB testing in a hospital setting (39%), when compared to those patients who completed the same computer‐based test remotely from their home (24%). While preliminary results, these observations suggest that the environment in which cognitive tests are performed may be a confounding factor. Despite the hospital setting being quiet and free of distractions, our findings suggest that levels of patient familiarity, anxiety, and comfort can alter performance and thus efficacy of cognitive testing.

In contrast to patients in the CODEX study, no patients enrolled in the COGNIGRAM study exhibited pre‐CI prior to undergoing a major joint arthroplasty procedure. Only 7 of 712 (1%) study participants showed evidence of major pre‐CI at baseline. The remarkable difference in the prevalence of pre‐CI in patients undergoing cardiac versus noncardiac procedures is incongruous with contemporary literature, where the incidence of pre‐CI in patients scheduled to undergo hip or knee replacement is 20%–32% (Culley et al., 2017; Evered et al., 2011; Silbert et al., 2015). The root causes underlying the incredibly low level of pre‐CI detected in our *COGNIGRAM* study population, compared to our *CODEX* population, are not entirely clear. However, we do note a significant difference in average ASA physical status classification score between patients in *COGNIGRAM* and *CODEX* (2.44 and 4.00, respectively). Level of education also showed a high degree of association with pre‐CI (Culley et al., 2017; Evered et al., 2011) and POCD (Moller et al., 1998; Rasmussen et al., 2003), and we report that only 86.3% of patients in *COGNIGRAM* had achieved educational attainment beyond high school versus 71.9% in *CODEX*. Finally, the study population of *COGNIGRAM* was, on average, 5 years younger than patients enrolled in similar studies and also differed with respect to several common comorbidities: In particular, we note a significantly lower rate of prior myocardial infarction (1.4%) and diabetes (5.3%) than previously reported (4.1% and 8.8.%, respectively) (Silbert et al., 2015).

Overall differences in the prevalence of pre‐CI cannot be explained by variance in the assessment methods as CBB was used in both studies. However, in *COGNIGRAM*, the testing interface was opened by a research assistant on a computer whereas with *CODEX*, it was opened through an email link on the computer or tablet. There were some minor differences in graphical appearance; however, the card‐based subtests were otherwise identical. General demographic differences such as the age of patient populations could be another factor (which will be determined when *CODEX* is completed). However, these discrepancies likely also point to inherent differences in the prevalence of risk factors in the study populations. For instance, the higher average incidence of pre‐CI in cardiac and vascular surgery patients may be attributed to the increased prevalence of cerebrovascular disease and chronic inflammation, both of which have a well‐described impact on cognition (Paradise & Sachdev, 2019; Silbert et al., 2007; Thunström et al., 2015; van der Flier et al., 2018). Ultimately, the specific incidence may be of secondary importance to an increased understanding that this condition is relatively common across all surgical populations.

Due to the ongoing nature of the *COGNIGRAM* and *CODEX* trials, data capture is not complete and prespecified analysis plans have yet to be conducted. However, observations from preliminary data suggest a discrepancy in education level between cardiac and noncardiac surgical populations, although not large enough to account for the disparity in pre‐CI between groups. Because the CBB is based on playing cards, memory, and response time, it should be less susceptible than other neurocognitive testing modalities to the effects of education level. This is an important area which requires further study, however, in order to elucidate whether the diagnostic thresholds of computerized tests like the CBB ought to be adjusted to reflect education level.

## 4 INTERPRETATION AND SIGNIFICANCE

Once both studies have been complete, the findings will ultimately have both direct and indirect impacts on patient care. In the short term, these parallel studies will identify pre‐CI in patients in our cardiac and noncardiac surgical populations who are at risk of postoperative cognitive complications. These patients can subsequently be prioritized for inclusion in multimodal preventative treatment programs, which are designed to reduce and prevent cognitive decline (Berger et al., 2018; Inouye et al., 2000; Needham et al., 2017; Sánchez et al., 2019). Our goal is to expand our prescreening approach to the entire surgical population (approximately 15,000 patients per year) at our tertiary center, Sunnybrook Health Sciences Centre (Toronto, ON).

Our work also demonstrates the feasibility of using computerized cognitive assessment tools like the CBB for large‐volume pre‐CI and P‐NCD screening. These tools are sensitive to minor changes in cognition and have been validated for use in a variety of clinical conditions, including following cardiac surgery (Maruff et al., 2013; Mielke et al., 2015; Silbert et al., 2004). Our unique application of preoperative cognitive testing will facilitate more rigorous perioperative cognitive screening and assessment in order to cultivate an awareness of pre‐CI and improve patient care. Notably, the portable and virtual nature of these tools allows for the conduct of remote assessments. This novel approach improves access to this service, especially for patients living in remote and rural regions. Furthermore, there is some evidence to suggest that computerized assessments are completed more quickly when done at home (Mielke et al., 2015), making it easier and more convenient for patients. Although the CBB has been partially validated in limited perioperative contexts (Ichimura et al., 2010; Silbert et al. 2004), there remains a paucity of robust studies to compare these modalities with conventional “pen and paper” tests in the preoperative period. Mounting evidence of the myriad advantages of computerized testing platforms presents a compelling reason to undertake such comparisons, however, which we offer as a future direction in perioperative neuroscience. In the long term, our findings will be made publicly available through publications and the activities of our Perioperative Brain Health Centre. We anticipate delivering long‐lasting benefits to the quality of life of surgical patients across Canada and worldwide. We offer a perspective that the detection and management of pre‐CI is of high priority in the critical care community: because it is not feasible to exclude all patients with pre‐CI from surgical candidacy. Our work forwards the hypotheses that the CBB may find application as a clinical tool for identifying condition‐NCD and that greater attention to preoperative cognition will allow for further refinements in our ability to identify and respond to pre‐CI as a sensitive predictor of P‐NCD.

## CONFLICT OF INTEREST

The authors have no conflicts of interest to disclose.

## AUTHOR CONTRIBUTIONS

CTAB, BAO, SA, AF, LK, and SC were all responsible for conception and design of this manuscript. CTAB, AF, and LK were responsible for data analysis. CTAB, AF, LK, and SC prepared the manuscript. All authors reviewed and approved the final manuscript.

### PEER REVIEW

The peer review history for this article is available at https://publons.com/publon/10.1002/brb3.2095.

## Data Availability

Data will be available upon request after publication of the primary results of each respective study by direct request to PI.
